# Supplementary material for: Identification of photoperiod-induced specific miRNAs in the adrenal glands of Sunite sheep (Ovis aries)
Source: Front Vet Sci. 2022 Jul 22;9:888207. doi: 10.3389/fvets.2022.888207 (PMC9354845; doi:10.3389/fvets.2022.888207)
Supplement: Supplementary file 1 [file Data_Sheet_1.ZIP › Supplementary file/miRDeep2 file.zip!/miRDeep2 file/Know_Structure/SPLP42A3/oar-mir-668.pdf]

miRBase precursor : oar-mir-668
Total read count : 30
oar-miR-668-5p read count : 3
oar-miR-668-3p read count : 26
remaining reads : 1

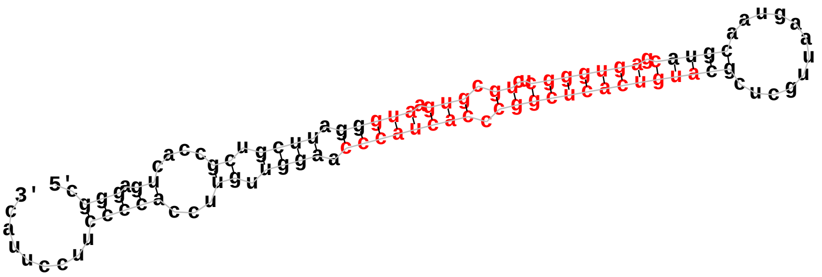

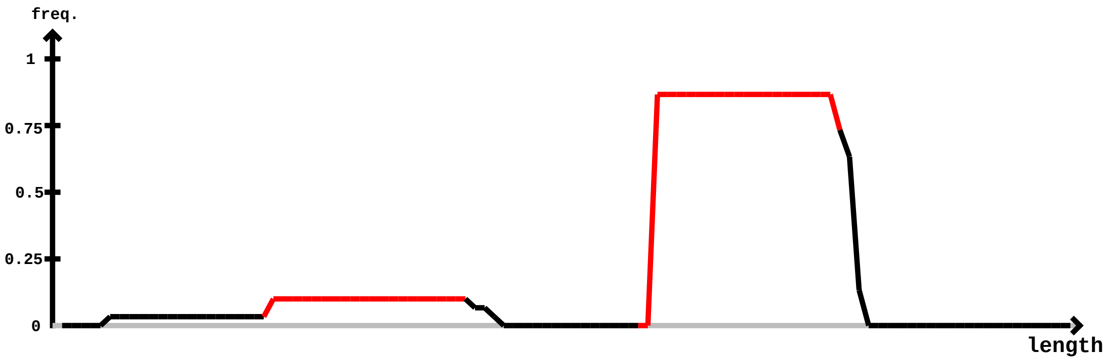


```
                                                                     oar-miR-668-3p
                                   oar-miR-668-5p
5'- cgggagucaccgcugcuuaggguaagugcgucucgggugagcaugcaaugaauugcucgcaugucacucggcccacuacccaagguuguuccaccccuuccuuac   -3'    exp
    .(((.((....((.((((.(((((.(((.((..(((((((.(((((............)))))))))))))).)))))))).)))).))...))))).........      reads    mm      sample
    ....agucaccgcugcuuagg....................................................................................      1        0       seq
    .....................guaagugcgucucgggugagc...............................................................      1        0       seq
    .....................guaagugcgucucgggugagcau.............................................................      1        0       seq
    .....................guaagugcgucucgggugagcaug............................................................      1        0       seq
    .............................................................ugucacucggcccacuacc.........................      4        0       seq
    .............................................................ugucacucggcccacuaccc........................      3        0       seq
    .............................................................ugucacucggcccacuaccca.......................      15       0       seq
    .............................................................ugucacucggcccacuacccaa......................      4        0       seq
```
